# Drug safety of systemic treatments for psoriasis: results from The German Psoriasis Registry PsoBest

**DOI:** 10.1007/s00403-015-1593-8

**Published:** 2015-09-10

**Authors:** K. Reich, U. Mrowietz, M. A. Radtke, D. Thaci, S. J. Rustenbach, C. Spehr, M. Augustin

**Affiliations:** Dermatologikum Hamburg, Hamburg, Germany; Department of Dermatology, Venereology and Allergy, Psoriasis Center, University Medical Center Schleswig-Holstein, Campus Kiel, Kiel, Germany; Institute for Health Services Research in Dermatology and Nursing (IVDP), University Medical Center Hamburg-Eppendorf, Hamburg, Germany; Excellence Center for Inflammation Medicine, University Medical Center Schleswig-Holstein, Campus Lübeck, Lübeck, Germany

**Keywords:** Drug safety, Pharmacovigilance, Psoriasis, Systemic treatment, Biologic treatment, Registry

## Abstract

The German Psoriasis Registry PsoBest was conducted in 2008 in order to investigate the long-term outcomes and safety of systemic treatments for moderate-to-severe psoriasis. Safety analysis of antipsoriatic drugs with special focus on serious adverse events (SAE) for infections, malignancies and major cardiac events (MACE) was done. Nationwide non-interventional patient treatment registry conducted in 251 active dermatology centers. Until June 2012, *n* = 2444 patients [40 % female; mean age 47.3 (SD 14.1) years; mean duration of disease 18.2 (SD 14.7) years] were recruited, including *n* = 1791 patients (3842 patient years) with conventional systemic drugs and *n* = 908 (3442 patient years) with biological drugs. Mean PASI (Psoriasis Area and Severity Index) at inclusion was 14.7, mean DLQI (Dermatology Life Quality Index) 11.1, mean BMI (Body Mass Index) 28.2. The overall rate of SAE per 100 patient years were 1.3 (SD 0.9) per 100 patient years in conventional systemic and 1.5 (SD 1.2) in biologics (*p* > 0.5, no significant difference). The rates per 100 patient years for single severe adverse events were as follows (systemic/biologics): serious infections, 0.33/0.65 [CI (confidence interval) 0.13–0.54/0.35–0.98]; MACE, 0.56/0.77 (CI 0.29–0.97/0.41–1.31); malignancies (except non-melanoma skin cancer), 0.46/0.49 (CI 0.22–0.84/0.21–0.97). There were no significant differences between single drugs in any of the safety parameters. The conventional systemic and biologic drugs for psoriasis show satisfying safety under routine psoriasis care in Germany with respect to infections, MACE and malignancies.

## Introduction

According to evidence-based guidelines, systemic drugs are treatments of choice for patients with moderate-to-severe psoriasis [13, 16]. In most countries, first-line systemic treatment includes methotrexate, ciclosporin A and retinoids, whereas the second-line treatment is based on the biologics infliximab, etanercept, ustekinumab and adalimumab for plaque type psoriasis and psoriatic arthritis, and golimumab for psoriatic arthritis. Only in Germany fumaric acid esters (FAE) are licensed for first-line treatment. Showing a chronic, persisting course of disease, psoriasis requires a long-term strategy for treatment over many decades [2, 3].

The German Psoriasis Registry PsoBest records safety, long-term efficacy, patient benefit and treatment regimens of psoriasis. Patients with moderate or severe psoriasis are included in PsoBest, when treatment with a conventional systemic agent or biologic is started for the first time. Observation time is extended to 10 years.

One of the major objectives of the patient registry PsoBest is the evaluation of safety and outcomes in systemic treatment of plaque type psoriasis and psoriatic arthritis [4]. The present analysis presents long-term safety outcomes from PsoBest with a special focus on severe infections, malignancies and major adverse cardiovascular events (MACE). The questions addressed were as follows:

1. How is the overall safety of conventional systemic drugs and biologics in the treatment of moderate-to-severe psoriasis and psoriatic arthritis?
2. In particular, are there any differences between the various treatments with respect to safety signals on severe infections, malignancies and MACE?

## Materials and methods

### Patient registry

All patients considered in the analysis were observed in The German Psoriasis Registry PsoBest [4]. This patient registry includes adult patients with moderate-to-severe psoriasis at the time point of a new drug to be started. The observation time for the patient is 10 years regardless of the treatment applied. Follow-up visits in the dermatology office are conducted in intervals of 3 months in the first half-year and every 6 months afterwards. In addition, 3 months after the physician visits, the patients are directly approached by mail for further information on the treatment status and patient reported outcomes. Patients without at least one follow-up visit are excluded from analysis, because of missing validation of therapy information. Patients were assigned to biologic cohort when they have been registered on adalimumab, etanercept, infliximab or ustekinumab. Starting a conventional systemic treatment with ciclosporin, fumaric acid ester or methotrexate, patients were referred to systemic treatment.

The outcomes measured in PsoBest follow the European consensus in the PsoNet network [14, 17, 18] and are thus harmonized with patient registries on psoriasis from other countries. Moreover, the assessment of safety has been adapted to the German registry on biologics and rheumatoid arthritis [19] and the international recommendations released in Europe [8, 11] and the United States [1, 10]. The registry conductance conforms to the German national guidance on patient registries [12]. In this guidance, an explicit set of requirements both on the study planning, conductance and data analysis is included. The quality assurance of the registry PsoBest follows the recommendations by controlling for structural, process and outcomes quality. The overall supervision of the standard operating procedures for PsoBest based on the guidances was provided by a DIN ISO 9001:2008 certification (certificate ID 170549705). Scientific quality is warranted by a scientific advisory board of German psoriasis experts. Regular annual investigator meetings and quarterly newsletters are provided in order to maintain high quality of investigator performance.

### Adverse events

In this analysis, only prospectively observed events were considered. Any event was classified serious (serious adverse event) in context of in-patient stay, life-threatening circumstances, neoplasms and death. All events observed were divided into 9 classes regarding infections, cardiovascular events and malignancies. Events, which are not matching any class, e.g., gastrointestinal disorders are not shown.

Infections were divided into ‘serious’—in the context of in-patient stay or life-threatening status, ‘severe’—with antibiotic prescription—and ‘non-severe’ including all other infections. Major adverse cardiovascular events are defined as irreversible events based on vascular obstruction comprising myocardial infarction, cardiac failure, cardiovascular death, acute coronary syndrome, hemiparesis ischemic stroke and cerebrovascular accident. To prevent loosing important information on cardiovascular events the category other cardiovascular events (right ventricular failure, coronary artery occlusion, cardiac arrest and myocarditis) was defined. Besides these groups no other events occurred regarding infections, cardiovascular events and malignancies. The event-class ‘malignancies’ comprises all neoplasms with additional regard to melanoma and non-melanoma skin cancer.

MedDRA (Medical Dictionary for Regulatory Activities) preferred terms for investigations as well as for surgical or medical procedures were only considered if they were single reported events, e.g., ‘tumor excision’ was only counted as event in malignancies if there was no tumor reported.

### Data analysis

The data analyzed in this study were gained in the time period from January 01, 2008 to December 31, 2012. The safety data were separately documented in MedDRA preferred terms for adverse events and severe adverse events.

The occurring events were attributed to the last treatment, applied with a 90-day window following the Manchester template. Only the events of malignancies and death were assigned to all previous systemic treatments, regardless of its exposure time. Events occurring within a combined treatment were assigned to all treatments as exposed.

The absolute number of events was recorded. All safety data reported were referred to exposure time (100 patient years). Confidence intervals were computed using inverse Chi-square distribution and significance level 0.05. Descriptive data comparisons were conducted with safety data from the international Psolar registry [15; Langley R et al. Malignancy Events in the Psoriasis Longitudinal Assessment and Registry (PSOLAR) Study: Current Status of Observations [unpublished poster presentation] EADV meeting: Prague; 2012; Leonardi C et al. Serious Infection Events in the Psoriasis Longitudinal Assessment and Registry (PSOLAR) Study: Current Status of Observations [unpublished poster presentation] EADV meeting: Prague; 2012; Naldi L et al. Major Adverse Cardiovascular Events (MACE) in The Psoriasis Longitudinal Assessment and Registry (PSOLAR) Study: Current Status of Observations [unpublished talk] EADV meeting: Prague 2012].

The statistical analysis was conducted with IBM SPSS Statistics for Microsoft Windows version 18. Analysis was performed for the groups systemic versus biologic and each single treatment regarding prior exposure to biologics and presence of concomitant conventional systemic therapies.

## Results

### Patient cohort

**Fig. 1:**
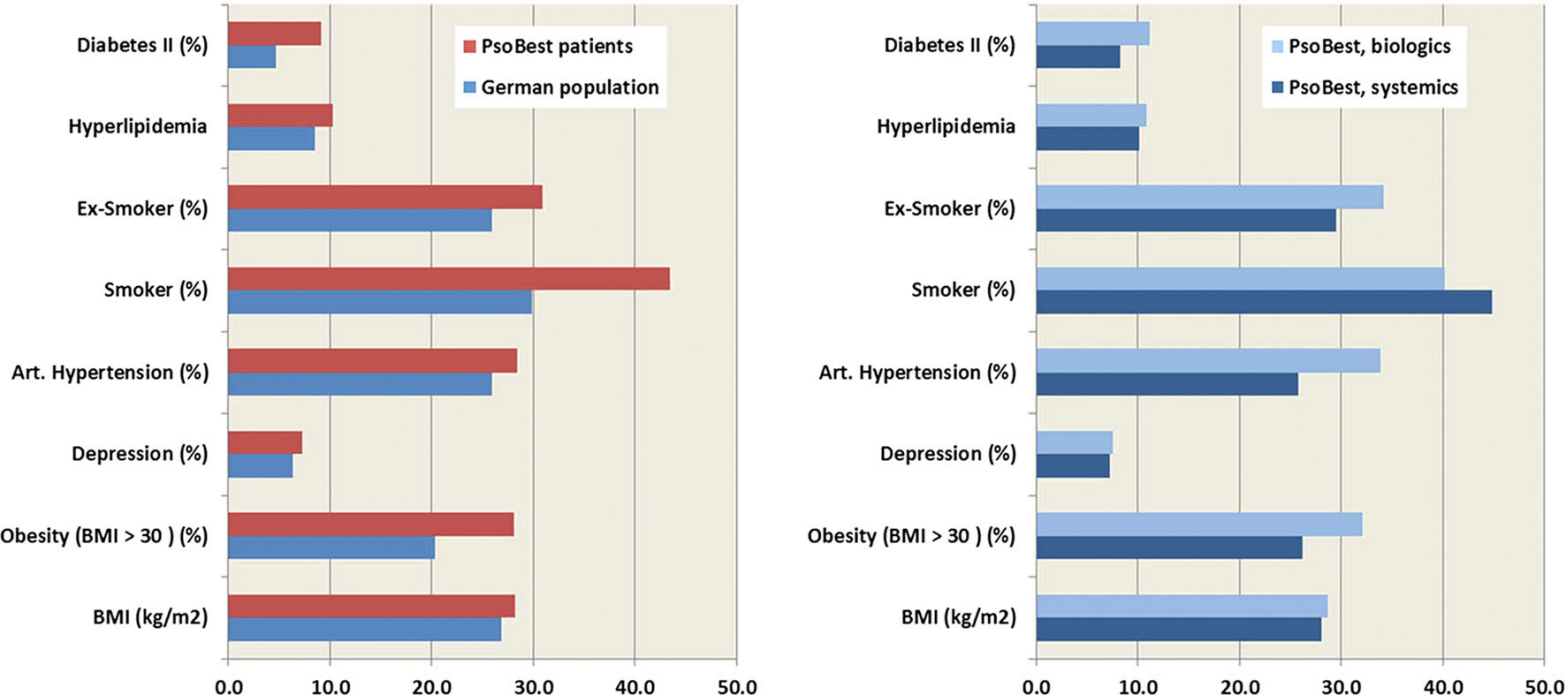
Rates for comorbidity in patients with psoriasis of the PsoBest registry compared to the age- and gender-adjusted rate of the German normal population (*left graph*) and comparison between patients started with biologics versus systemic (*right* *graph*; *n* = 2444)

In total, 2444 patients (40 % women; 634 patients on biologic, 1584 on conventional systemic treatment; 266 patients without qualifying treatment) were included in the analysis. Mean age was 47.4 (SD 14.1) years, the mean duration of disease 18.2 (SD 14.7) years (Table 1). There is a total exposure time to biologics of 1463 years and 1733 to conventional systemic treatments (Table 2). In total, there was a significant rate of comorbidity and co-medication in these patients compared to patients without psoriasis (Fig. 1), indicating a higher risk for adverse events in patients with psoriasis. Moreover, patients receiving biologics showed significantly higher rates of relevant comorbidities such as cardiovascular disease, obesity and diabetes (Fig. 1).

**Table 1:** Clinical patient characteristics of The German Psoriasis Registry PsoBest at baseline and number of adverse events and serious adverse events in the observation time

	Total (2444 patients registered)		Biologic treatment (634 patients)		Systemic treatment (1584 patients)	
	Number	%	Number	%	Number	%
Female	975	39.9	230	36.3	651	41.1
Male	1.469	60.1	404	63.7	933	58.9
Psoriasis–arthritis	506	20.7	230	36.3	223	14.1

	Total (2444 patients registered)					Biologic treatment (634 patients)					Systemic treatment (1584 patients)				
	Mean	Min	Max	SD	Valid measures	Mean	Min	Max	SD	Valid measures	Mean	Min	Max	SD	Valid measures
Age (years)	47.4	18.0	88.0	14.1	2438	48.0	18.0	87.0	13.3	633	46.8	18.0	88.0	14.4	1580
BMI	28.2	14.7	63.3	5.8	2430	28.7	15.0	54.0	5.9	629	28.0	14.7	63.3	5.8	1575
Waist–hip ratio*	0.9	0.6	1.4	0.1	2299	0.9	0.6	1.4	0.1	593	0.9	0.6	1.3	0.1	1498
PASI	14.7	0.0	64.9	9.7	2388	15.1	0.0	64.9	10.3	628	14.4	0.0	64.8	9.5	1544
DLQI	11.2	0.0	30.0	7.1	2395	11.6	0.0	30.0	7.5	621	10.9	0.0	30.0	6.8	1556
BSA	24.0	0.0	100.0	20.5	2379	24.3	0.0	100.0	21.0	620	22.9	0.0	100.0	19.6	1545
Duration of psoriasis (years)**	18.2	0.0	74.0	14.1	2312	21.9	0.0	62.0	14.1	601	16.9	0.0	74.0	14.0	1500
Observation time in registry (m)**	16.5	0.0	58.0	15.7	2444	22.0	0.0	58.0	16.2	634	14.5	0.0	55.9	14.7	1584
Number of adverse events	1.1	0.0	14.0	1.8	2444	1.1	0.0	13.0	1.7	634	1.1	0.0	14.0	1.9	1584
Number of serious adverse events	0.1	0.0	8.0	0.5	2444	0.2	0.0	4.0	0.5	634	0.1	0.0	8.0	0.5	1584

Differences between treatment groups are marked

* *p* ≤ 0.01, ** *p* ≤ 0.001

**Table 2:** Number of exposed patients and treatment periods including mean and total treatment time

Exposition	Patients	Periods	Exposure time per period (m)				Total exposure time (years)
			Mean	Min	Max	SD	
Adalimumab	462	480	15.6	0.0	55.3	14.2	623.0
Etanercept	298	320	13.5	0.0	52.5	13.0	360.5
Infliximab	108	109	14.1	0.0	48.5	12.1	127.9
Ustekinumab	257	264	16.0	0.0	44.4	13.3	351.8
Anti-TNF	756	909	14.7	0.0	55.3	13.6	1111.4
Biologics	908	1173	15.0	0.0	55.3	13.5	1463.3
Cyclosporine	229	246	7.8	0.0	50.6	9.4	160.0
FAE	981	1030	9.4	0.0	55.8	11.7	807.8
Methotrexate	798	861	10.7	0.0	54.5	12.1	765.2
Systemics	1791	2137	9.7	0.0	55.8	11.6	1733.0

### Drug safety

#### Overall rate of serious adverse events

The overall rate of SAE was 1.3 (SD 0.9) per 100 patient years in systemic and 1.5 (SD 1.2) in biologics (*p* > 0.5, no significant difference).

#### Rate of infections

**Fig. 2:**
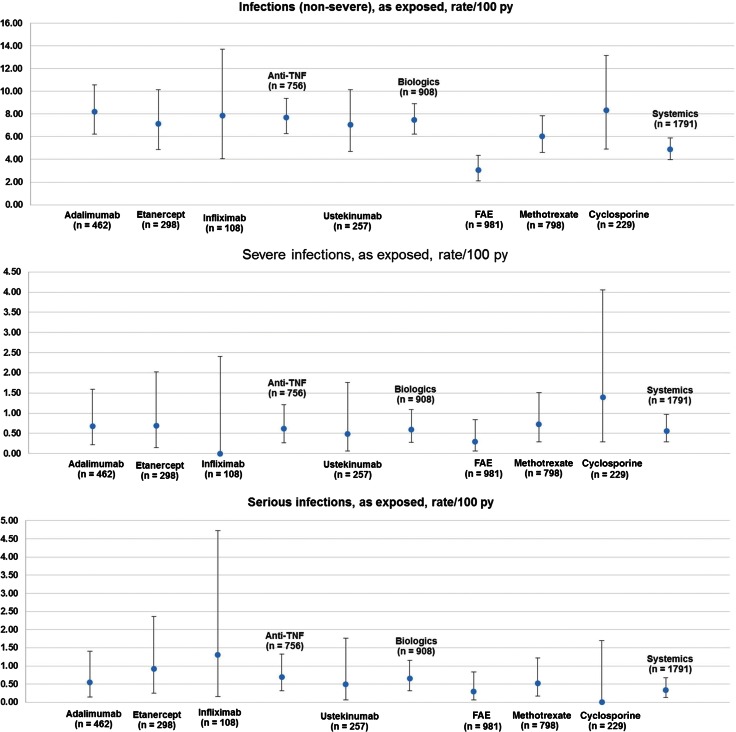
Rates per 100 patient years of infections (non-severe, severe and serious) in psoriasis patients with systemic and biological drugs (*n* = 2444), *bars* show confidence interval

The rate for serious infections was 0.33 (SD 0.20) per 100 patient years in systemic treatments and 0.65 (SD 0.33) in biologic treatments (*p* > 0.05, no significant difference; Fig. 2). Rates of 0.56 (SD 0.27) and 0.59 (SD 0.31) were observed for severe infections and rates for non-severe infections resulted in 4.88 (SD 0.89) in systemic and 7.50 (SD 1.25) in biologics. There were no significant differences between the status of previous exposure to biologics or concomitant conventional systemic therapy in biologic treatments. Patients with previous exposure to biologics receiving systemic therapies had a higher risk for non-severe infections (12.5 versus 4.5 regarding previous biologics, 12.2 versus 5.4 regarding concomitant systemic, *p* < 0.05).

#### Major cardiac adverse event

**Fig. 3:**
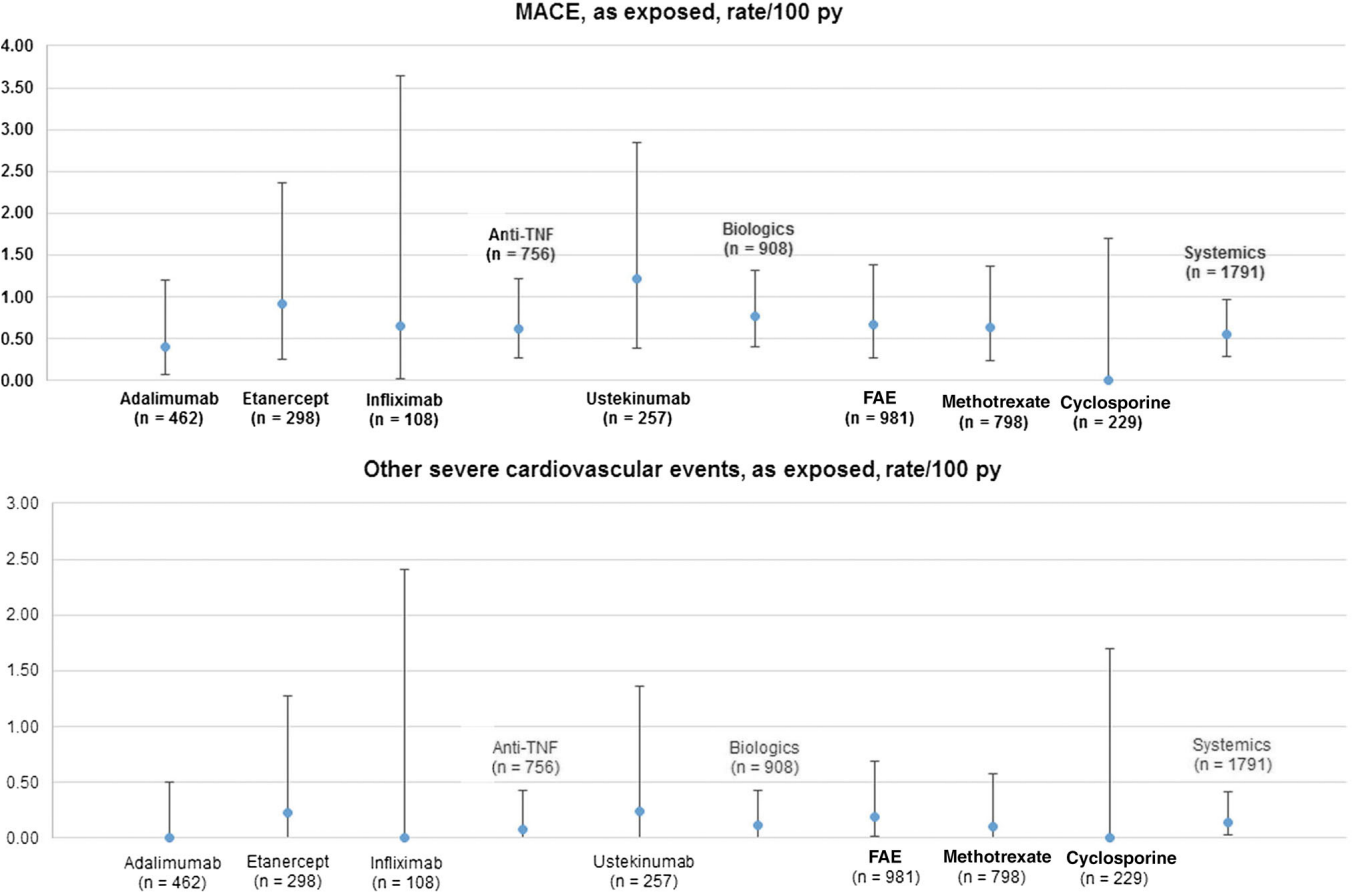
Rates per 100 patient years of MACE (major cardiac events) and other severe cardiovascular events in psoriasis patients with systemic and biological drugs (*n* = 2444), *bars* show confidence interval

The MACE rate did not significantly differ between conventional and biologic treatments (0.56 (SD 0.27) versus 0.77 (SD 0.36) per 100 patient years) (Fig. 3). Also, there were no significant differences between different single drugs. Similarly, the rate of other severe cardiovascular events did not significantly differ between groups.

#### Rate of malignancies

**Fig. 4:**
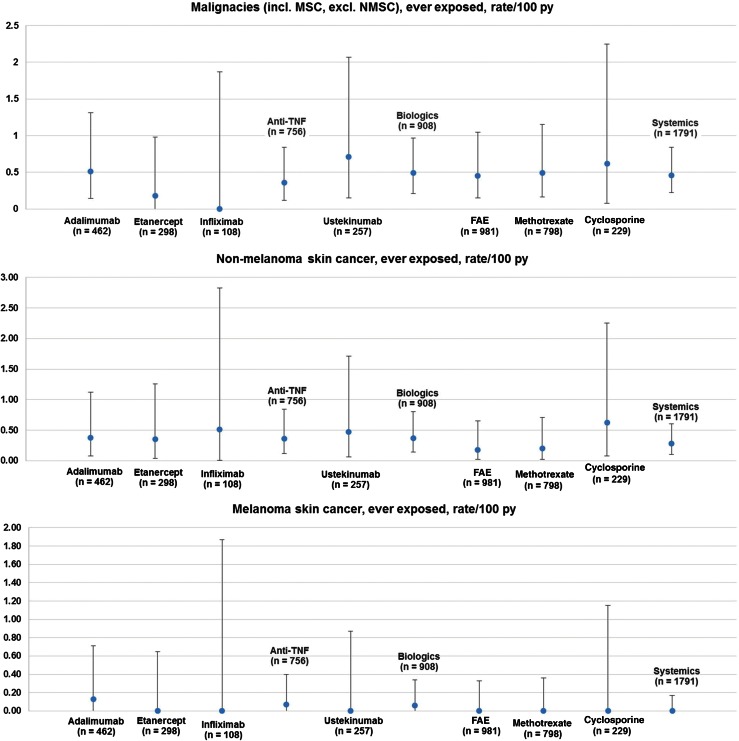
Rates of malignancies in psoriasis patients with systemic and biological drugs, including all malignancies except non-melanoma skin cancer (*top*), non-melanoma skin cancer (*middle*), and melanoma skin cancer (*bottom*); *n* = 2444, *bars* show confidence interval

The overall rate of malignancies (except NMSC) per 100 patient years was 0.46 (SD 0.24) in patients receiving systemic and 0.49 (SD 0.28) in patients receiving biologics (*p* > 0.5, no significant differences, *n* = 2444); Fig. 4. There were no relevant differences between any drugs with respect to “all malignancies except non-melanoma skin cancer”, “non-melanoma skin cancer” and “melanoma skin cancer”.

## Discussion

Patient treatment registries are large databases reflecting real-world and long-term courses of disease. For psoriasis a series of national registries with comparable data sets has been established in Europe, in order to gain robust data on safety, tolerability and outcomes of systemic drugs including biologics. The present data analysis from The German Psoriasis Registry PsoBest intended to gain first safety information on antipsoriatic drugs in German routine care. Baseline data indicate that patients in the registry could have a higher risk for cardiovascular complications since comorbidity rates are elevated when compared to age-adjusted population-based rates. This potential bias needs to be considered when analyzing any registry data for safety. If compared to the overall cohort of psoriasis patients, selection events in the registries may occur due to variations in the access to treatments within a country or—even more—between countries [9]; e.g., it is remarkable that in most countries the proportion of women receiving biologics (but not systemic) is much lower than of men. The results of recent publications of psoriasis registries from different countries, however, are in line with our findings, suggesting no increased risk of serious or fatal AE in biologics compared to conventional systemics [5, 7].

Further limitations of registries derive from the fact that there is no random assignment of treatments. Thus, different treatment groups may show structural inequalities, which can confound results. For this, direct comparisons between drugs are limited, if not adjusted for the inhomogeneities. In the current analysis, no specific adjustments for baseline differences were performed, since the unadjusted outcomes indicate very low rates of safety signals across all treatment arms. Another limiting factor is the limited number of patients included in the analysis. Thus, small differences and rare events may have been missed. Since the inclusions into the registry are ongoing, data analyses will be conducted repeatedly. With respect to generalizability of the data, it needs to be taken into account that the current safety outcomes on patients with moderate-to-severe psoriasis may not be transferable to patients exposed to these drugs with other diseases; e.g., it has been shown that there is a different safety profile for different rheumatologic diseases [6]. Overall, these limitations which are mostly inherent to patient safety registries need to be considered. In contrast, the data’s strength is the non-selective character providing external validity and the systematic approach of nationwide solicited real-world safety data acquisition.

In total, with respect to safety signals, there have not been observed any indications for elevated risks of using systemic or biologic drugs for psoriasis in Germany. When compared to international data, like the Psolar registry—which mostly recruited patients in North America—the rates for the safety indicators are in the same range. For example, overall rates for neoplasms were 0.5/0.5 for systemic/biologics in PsoBest and 0.6/0.6 in Psolar, all data related to 100 patient years. Similarly, rates for all MACE were 1.0/0.8 in PsoBest and 1.1/1.3 in Psolar. Greater differences, but still on a low level, were found for the rates of overall severe infections (0.6/0.6 in PsoBest and 1.6/1.2 in Psolar). These differences may derive from variations in attributing infection events to non-severe, severe or serious types.

In conclusion, this analysis from The German Psoriasis Registry PsoBest confirms pharmacovigilance data from other registries, indicating a satisfying safety of the systemic and biological drugs used in Germany for moderate-to-severe psoriasis.
